# Supplementary figures and images for: In eubacteria, unlike eukaryotes, there is no evidence for selection favouring fail-safe 3’ additional stop codons
Source: PLoS Genet. 2019 Sep 17;15(9):e1008386. doi: 10.1371/journal.pgen.1008386 (PMC6764699; doi:10.1371/journal.pgen.1008386)

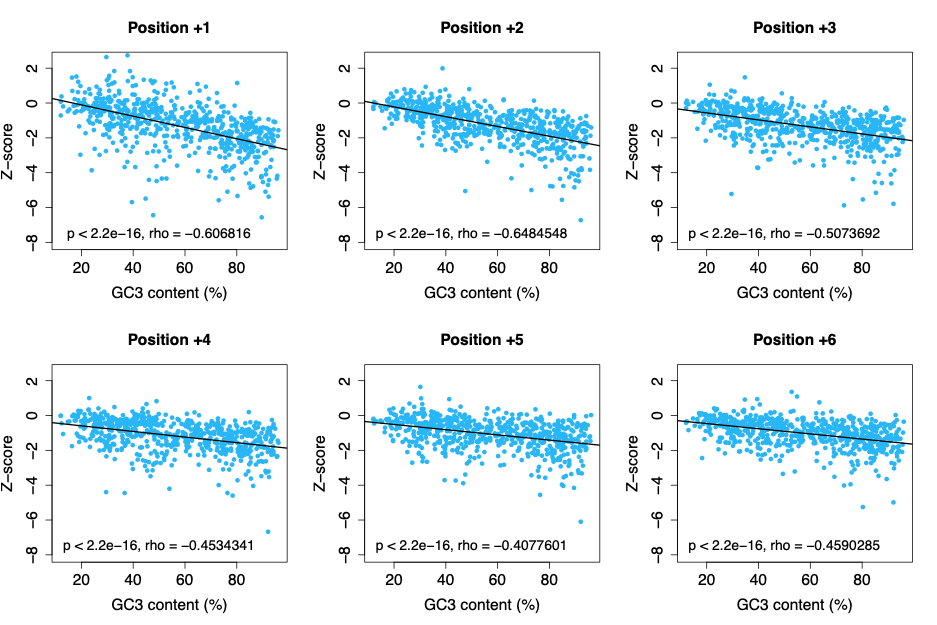

Supplement: S1 Fig — Significant negative relationships were observed between Z-score and genomic GC3 content at each position (Spearman’s rank: p < 2.2 x 10^−16^ for all positions; ρ = -0.61 at position +1, ρ = -0.65 at position +2, ρ = -0.51 at position +3, ρ = -0.45 at position +4, ρ = -0.41 at position +5, ρ = -0.46 at position +6). (TIF) [file pgen.1008386.s001.tif]

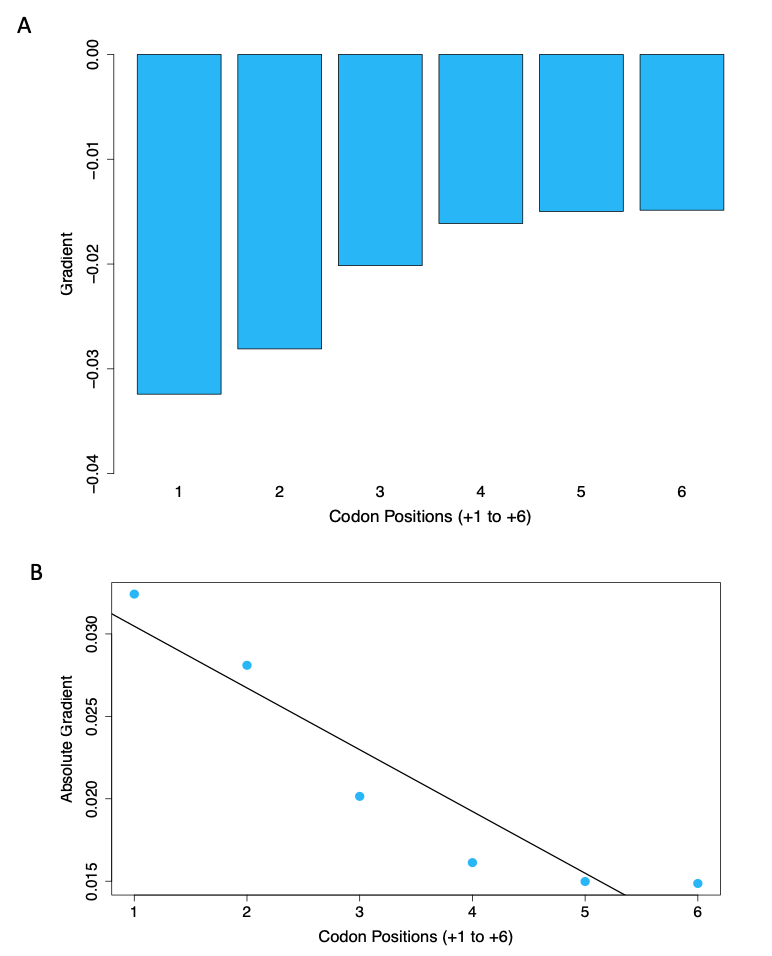

Supplement: S2 Fig — Raw gradients (of Z-score plotted against genomic GC3) plotted at each of the six positions downstream (A). Absolute gradients plotted against codon position (B). Our expectation under the fail-safe hypothesis is that at codon position +1, stops will be largely resistant to GC pressure while at position +6 this resilience will be diminished. We thus predict that looking across genomes, the plot of ASC usage against GC content should be flatter at site +1 than at site +6. Interestingly, there is a significant correlation between absolute gradient and distance from the primary stop (Spearman’s rank: p = 2.8 x 10^−3^ ρ = -1). We therefore infer that either the presence of ASCs is more resilient to GC pressure when located further downstream relative to the primary stop, or ASCs are actively selected against at positions closest to the primary stop. Both of these inferences go against the fail-safe hypothesis. (TIF) [file pgen.1008386.s002.tif]

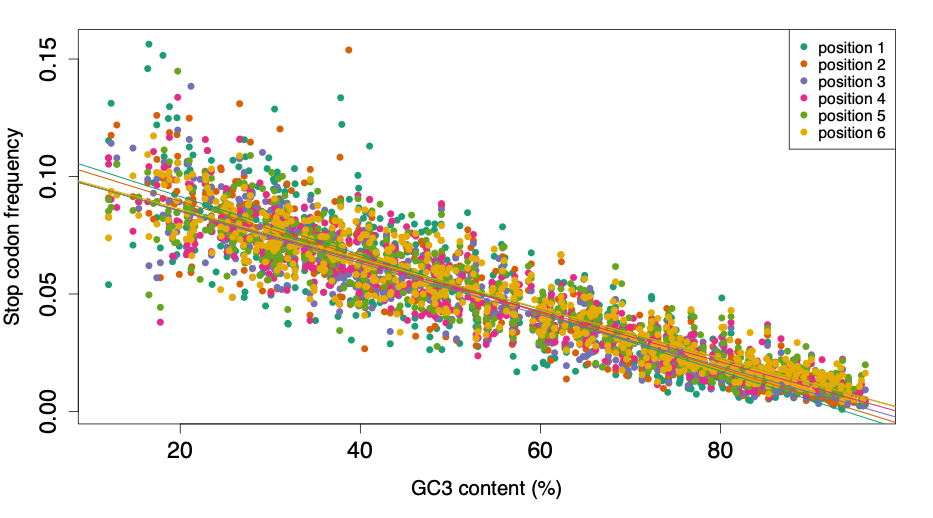

Supplement: S3 Fig — There is a significant negative correlation between the variables at all positions tested (Spearman’s rank: p < 2.2 x 10^−16^ for all positions; ρ = -0.92 for position +1, ρ = -0.95 for position +2, ρ = -0.95 for position +3, ρ = -0.94 for position +4, ρ = -0.93 for position +5, ρ = -0.94 for position +6). The gradient of the linear model fitted for position +1 is significantly different than that of position +6 (p = 2.035732 x 10−10), with position +6 having the more negative gradient. (TIF) [file pgen.1008386.s003.tif]

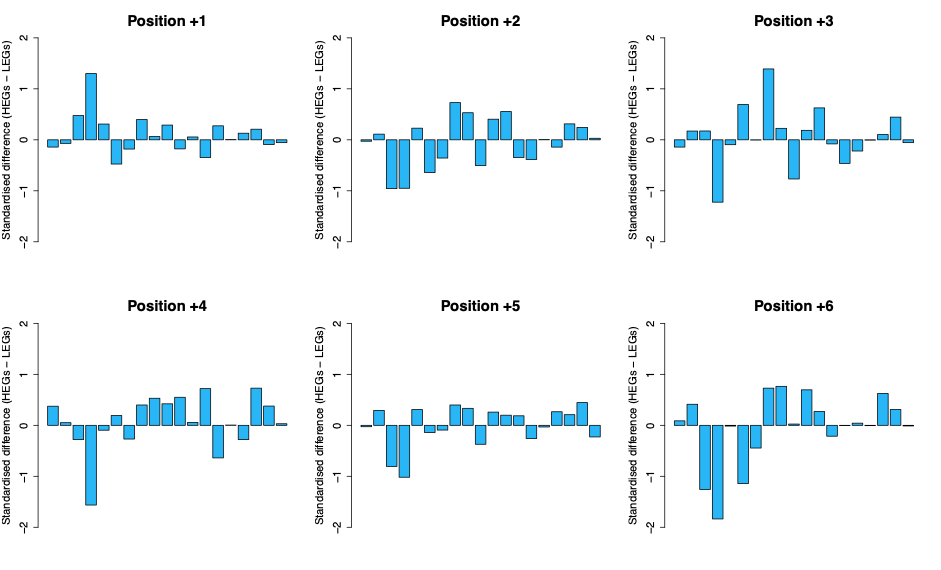

Supplement: S4 Fig — Each bar represents one genome, with genomes ordered by genomic GC3 content from left to right. Bar heights represents the raw difference between in ASC frequency between the two groups tested. A positive difference represents enrichment in the HEGs group, a negative difference represents enrichment in the LEGs group. There is a no significant difference between HEGs and LEGs at any position (Wilcoxon signed-rank test: p > 0.05/6). (TIF) [file pgen.1008386.s004.tif]

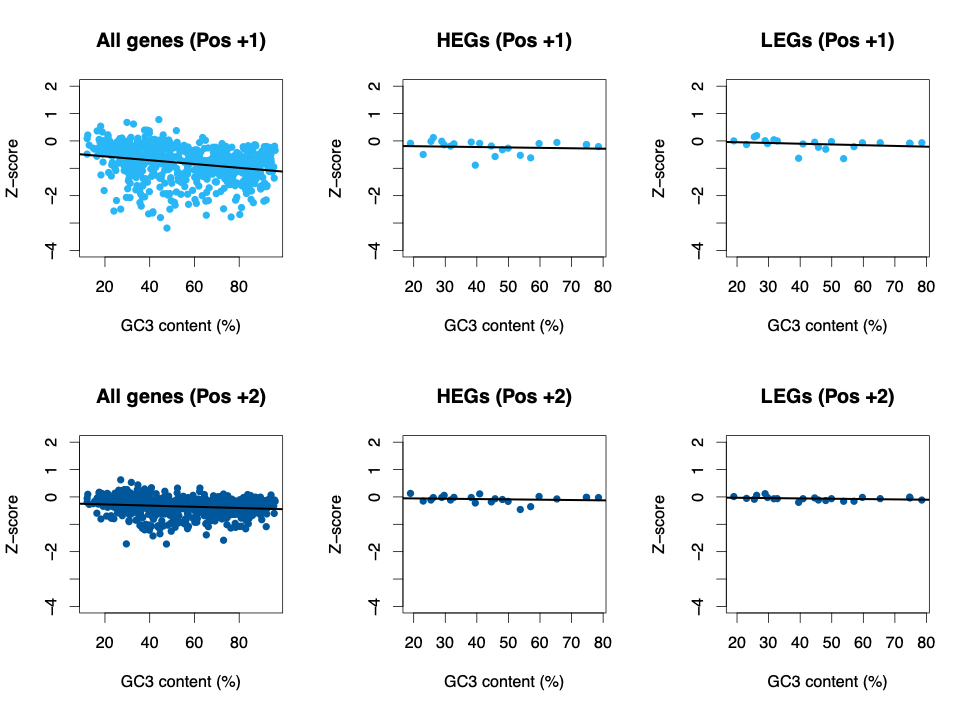

Supplement: S5 Fig — Only position +1 and position +2 are considered as these are the only sites where a signal for ASC enrichment has been noted. We find Z-scores to be negatively correlated with genomic GC3 when considering all genes at position +1 (Spearman’s rank: ρ = -0.3054869, p < 2.2 x 10^−16^) and position +2 (Spearman’s rank: ρ = -0.1880088, p = 1.62 x 10^−06^). There is no relationship between Z-score and genomic GC3 in HEGs or LEGs at either position (Spearman’s rank, p > 0.05). (TIF) [file pgen.1008386.s005.tif]

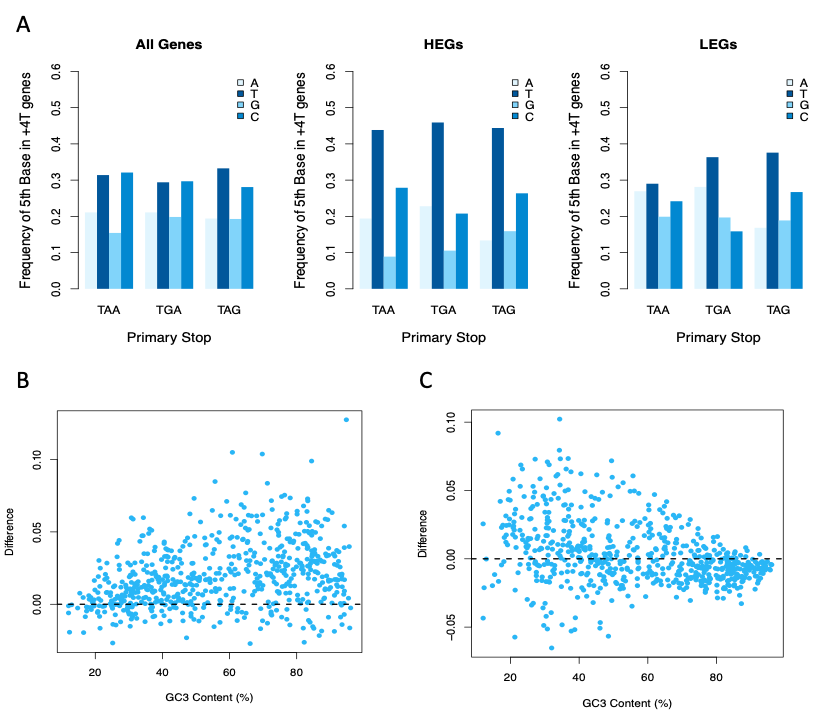

Supplement: S6 Fig — Fifth site nucleotide frequencies in +4T-containing genes of different primary stop and expression level (A). Frequencies of TC (B) and TT-starting (C) codons at position +1 compared to the average frequency of the respective codons between positions +1 to +6. Positive scores represent enrichment whilst negative scores represent under-representation. (TIF) [file pgen.1008386.s006.tif]

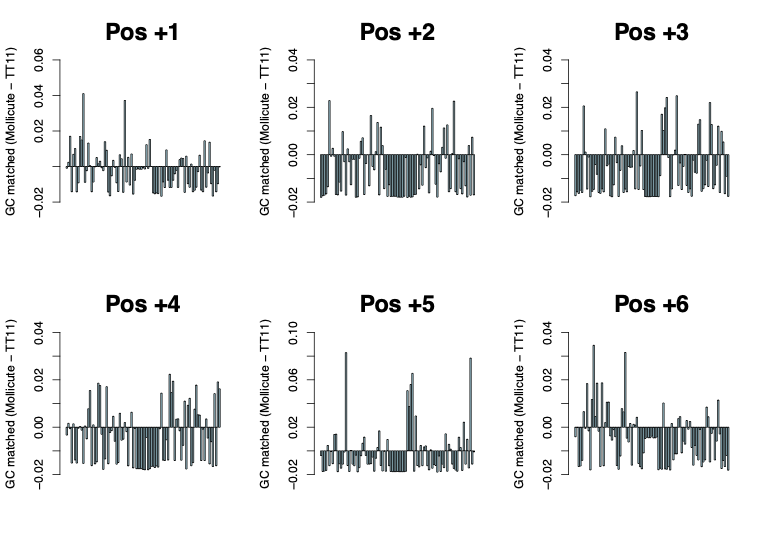

Supplement: S7 Fig — Each bar represents the frequency difference between a mollicute genome and the average of its GC-matched TT11 genomes. TGA was underrepresented at positions +3 and +5 only (Wilcoxon signed-rank tests: p = 0.11 for position +1; p = 0.15 for position +2; p = 1.5 x 10^−3^ for position +3; p = 0.70 for position +4; p = 6.8 x 10^−4^ for position +5; p = 0.11 for position +6). (TIF) [file pgen.1008386.s007.tif]

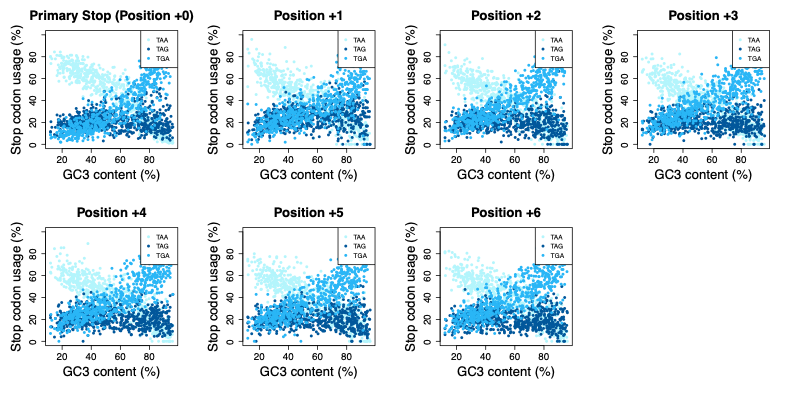

Supplement: S8 Fig — Contra to our expectations, we find codon usage at positions +1 to +6 to be consistent with that of the primary stop. (TIF) [file pgen.1008386.s008.tif]

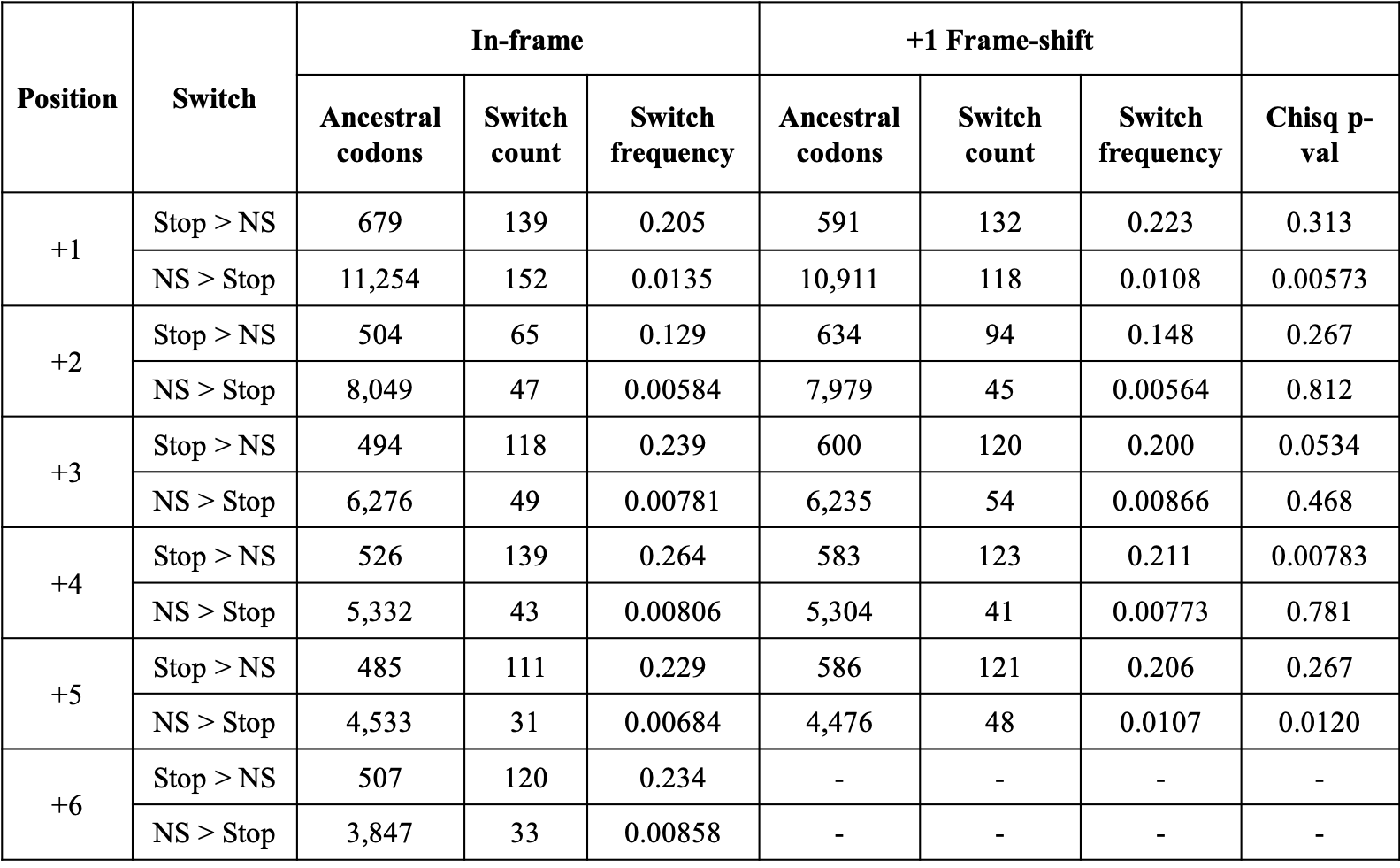

Supplement: S1 Table — (TIF) [file pgen.1008386.s009.tif]

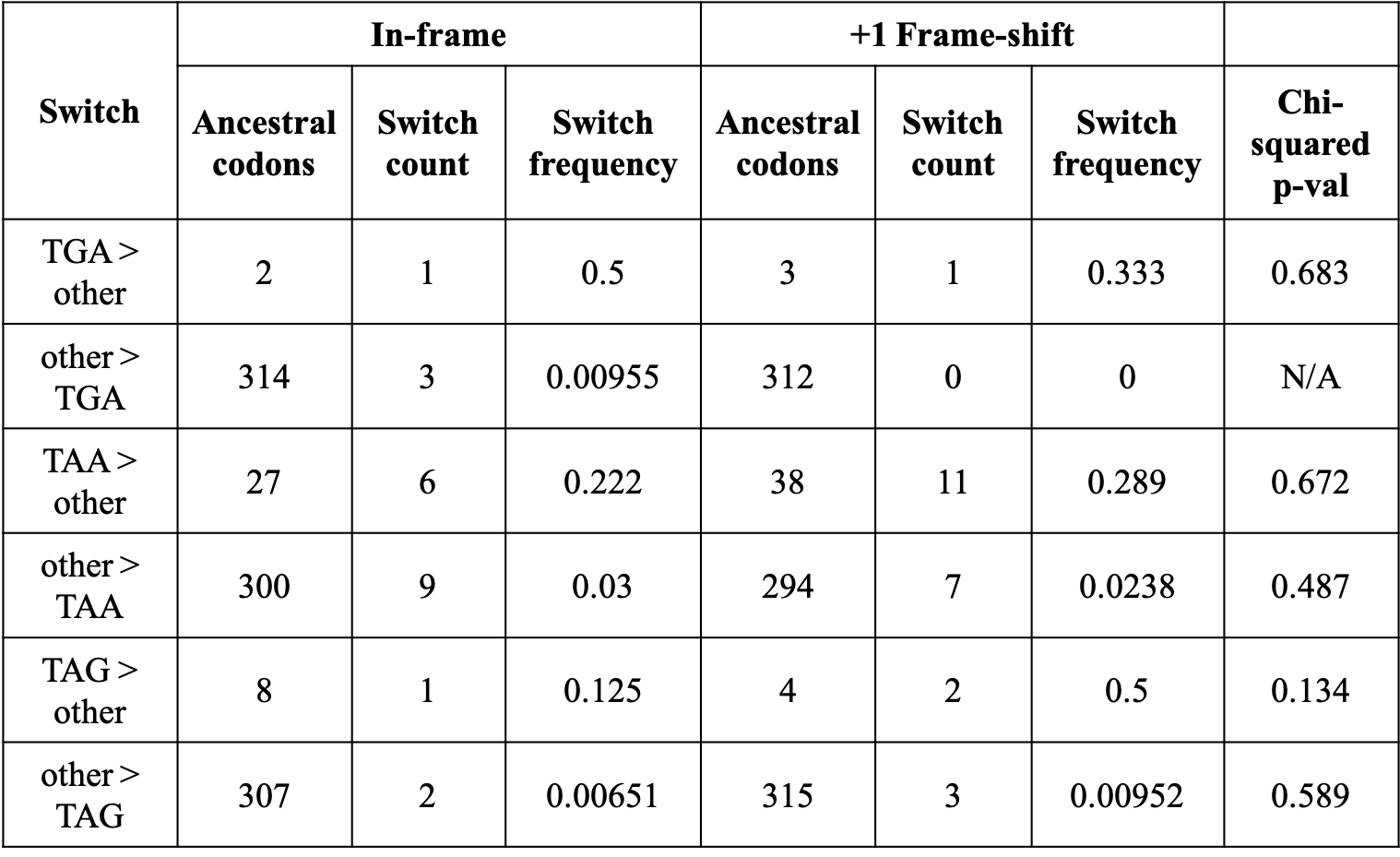

Supplement: S2 Table — (TIF) [file pgen.1008386.s010.tif]

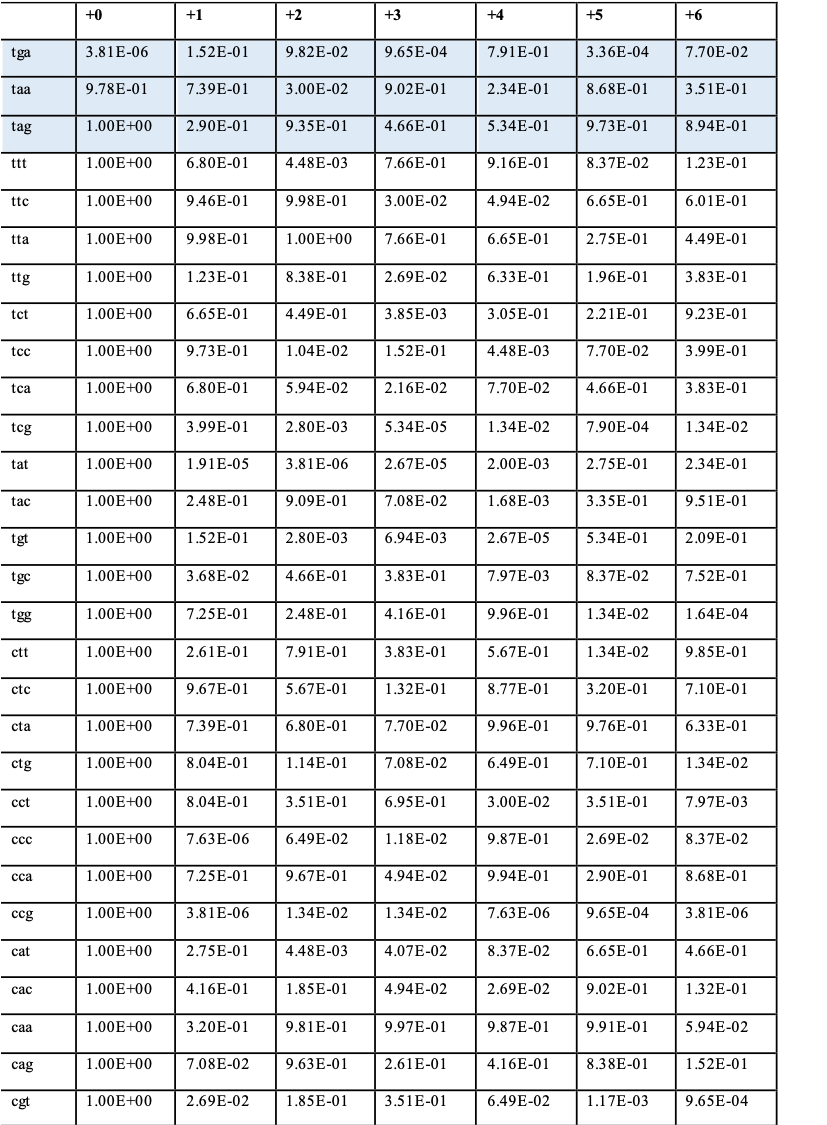

Supplement: S3 Table — Observed frequencies were compared to predicted frequencies using one-tailed Wilcoxon-signed rank tests, the p-values from which are found in the table below. A significant p-value represents significant under-enrichment in the TT4 genomes. Stop codons are highlighted in blue. (TIF) [file pgen.1008386.s011.tif]

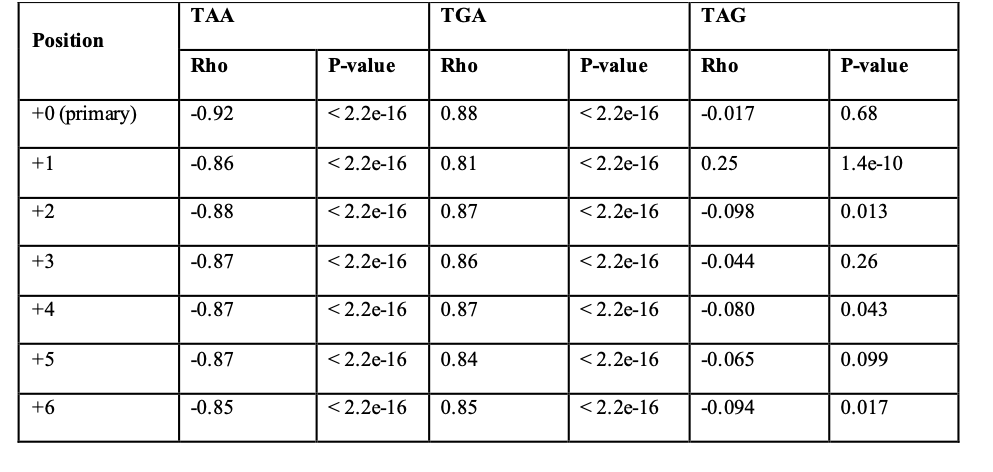

Supplement: S4 Table — (TIF) [file pgen.1008386.s012.tif]

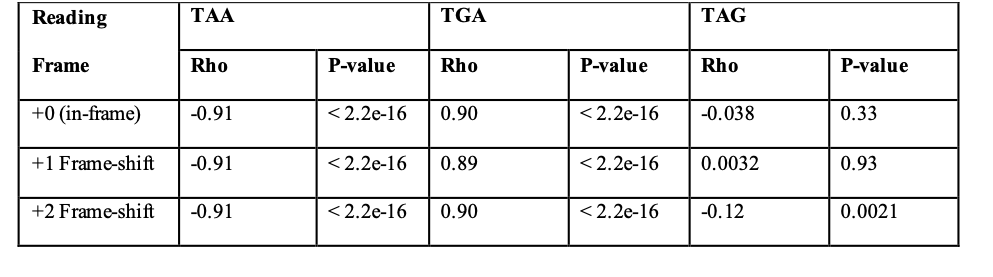

Supplement: S5 Table — (TIF) [file pgen.1008386.s013.tif]

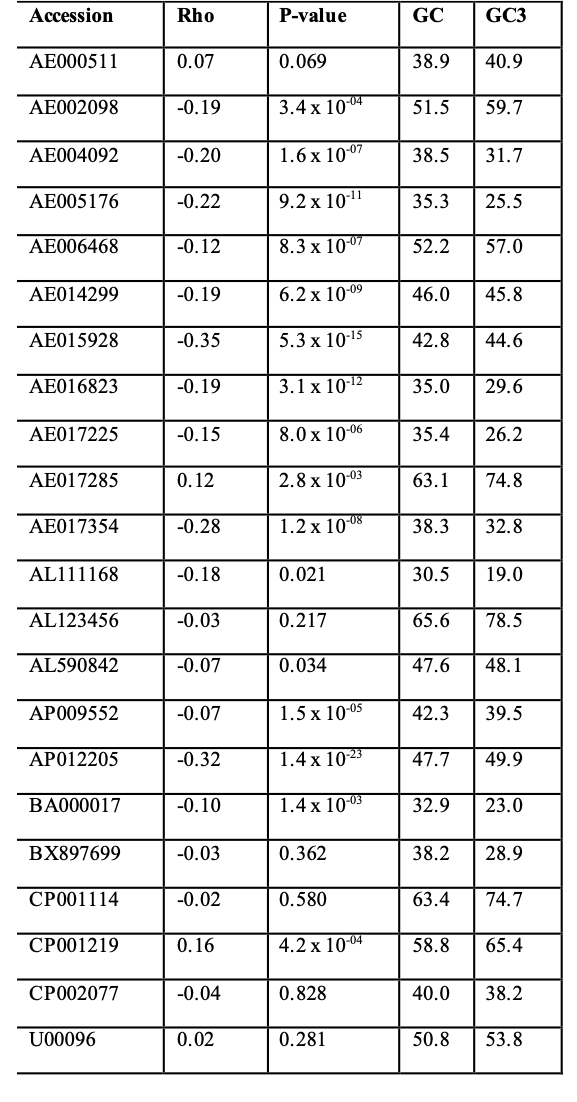

Supplement: S6 Table — Gene expression (represented by experimental protein abundance data) and gene nucleotide lengths were used in Spearman’s rank tests. (TIF) [file pgen.1008386.s014.tif]

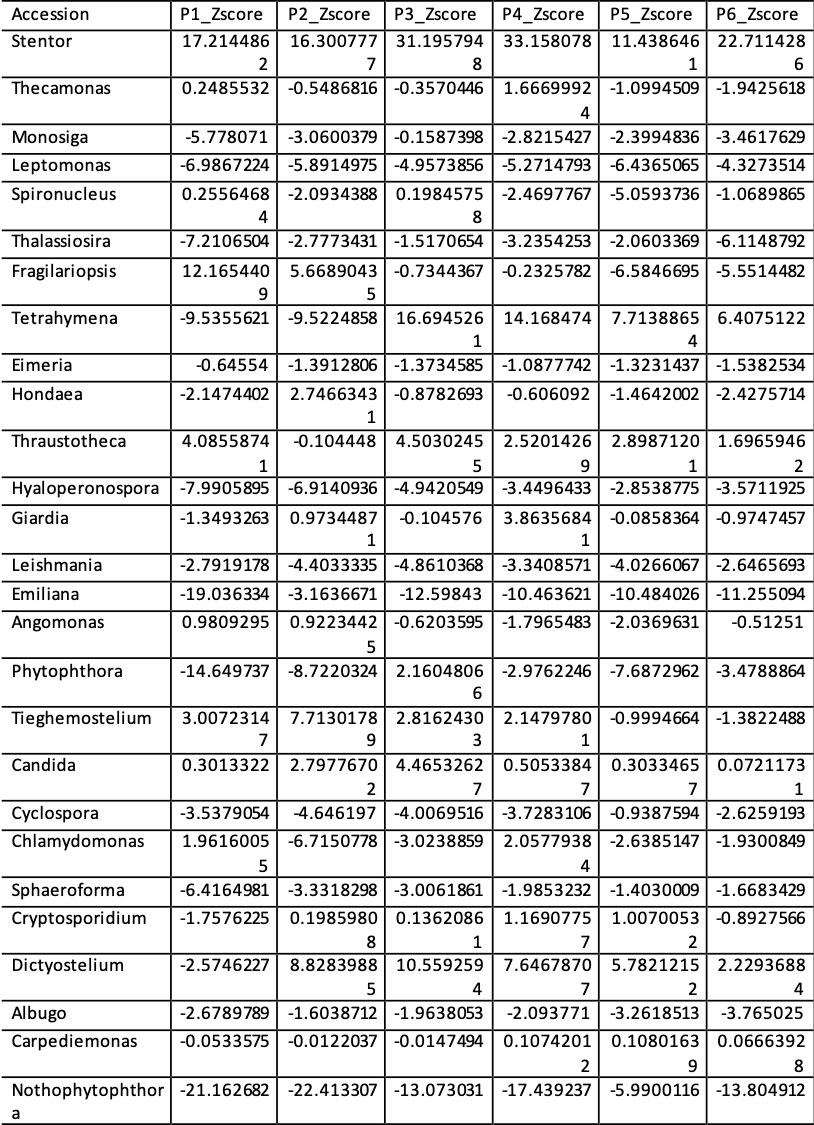

Supplement: S7 Table — Z-scores represent deviation in ASC frequency from dinucleotide-controlled simulations. (TIF) [file pgen.1008386.s015.tif]

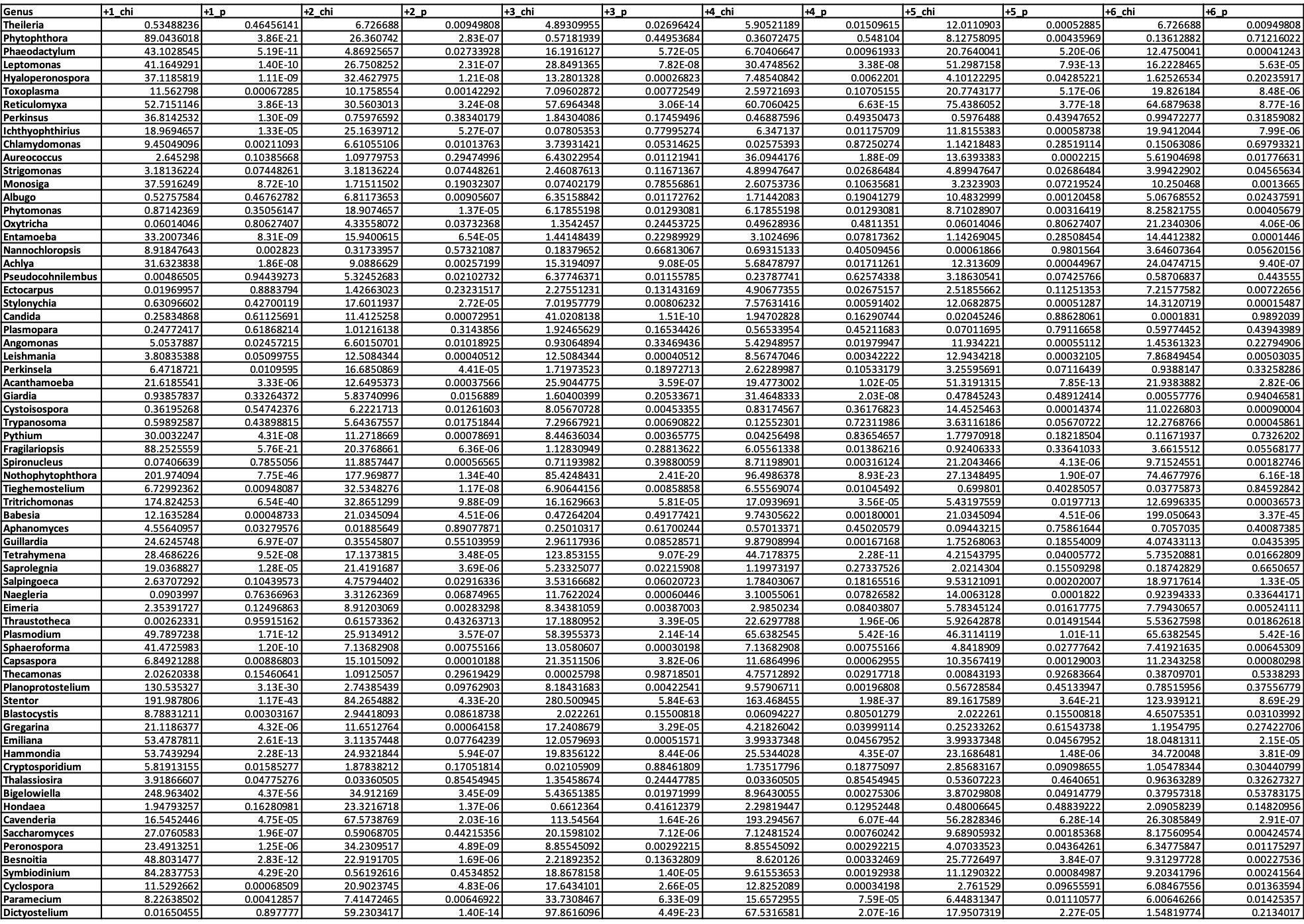

Supplement: S8 Table — (TIF) [file pgen.1008386.s016.tif]

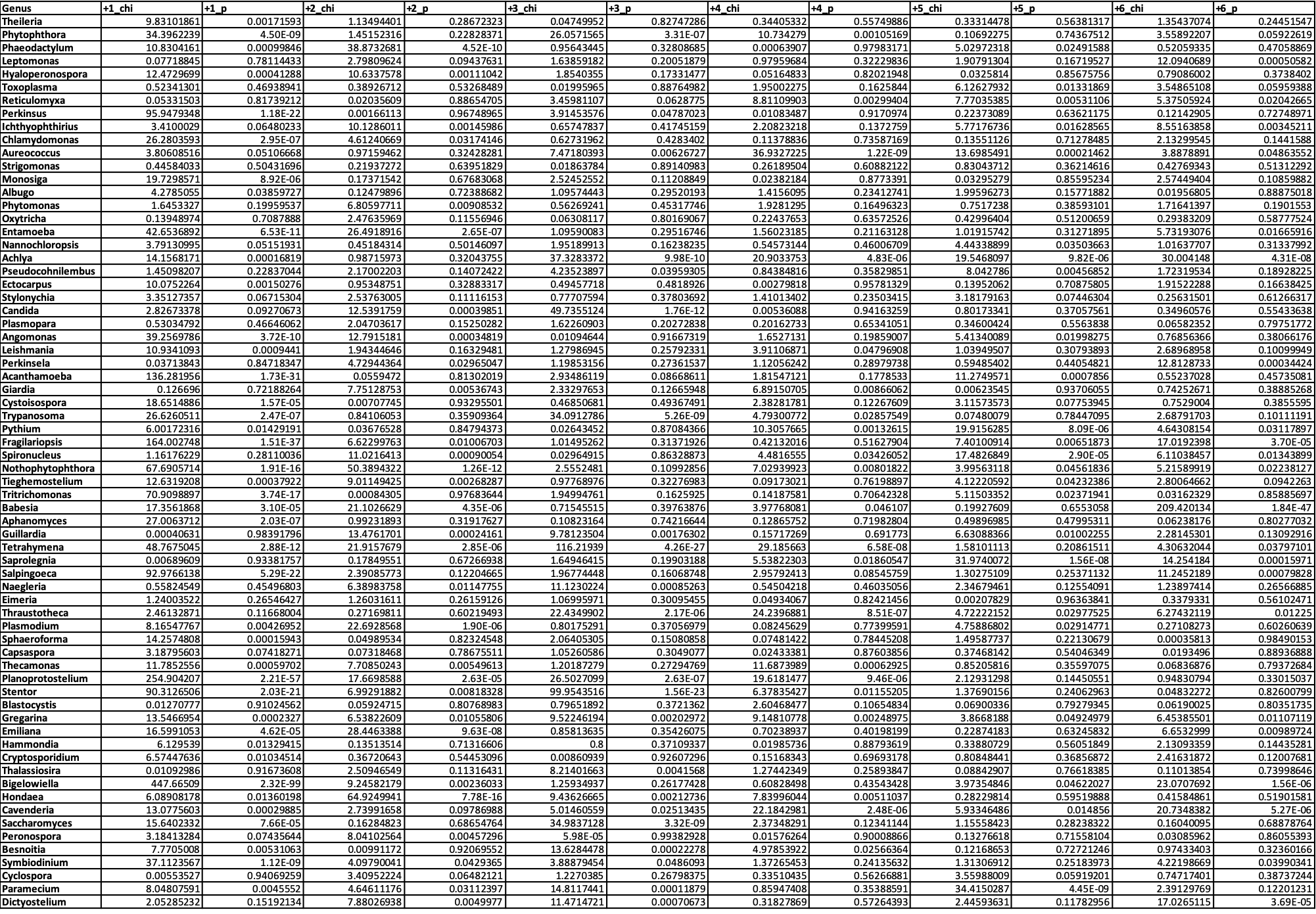

Supplement: S9 Table — (TIF) [file pgen.1008386.s017.tif]
