# Supplementary material for: In eubacteria, unlike eukaryotes, there is no evidence for selection favouring fail-safe 3’ additional stop codons
Source: PLoS Genet. 2019 Sep 17;15(9):e1008386. doi: 10.1371/journal.pgen.1008386 (PMC6764699; doi:10.1371/journal.pgen.1008386)
Supplement: S1 Text — (DOCX) [file pgen.1008386.s018.docx]

**S1 Text. Supporting text for S1 Fig, S2 Fig, and S3 Fig.**

A prediction of the fail-safe hypothesis is that the closer a site is to the primary stop codon, the stronger the selection should be to preserve an ASC (all else being equal), closer stops having the effect of reducing costs of error prone readthrough. One way to test this hypothesis is to consider the extent to which ASC frequency is resilient to underlying GC pressure. Our expectation under the fail-safe hypothesis is that at codon position +1, stops will be largely resistant to GC pressure while at position +6 this resilience will be relatively diminished. We thus predict that looking across genomes, the plot of ASC usage against GC content should be flatter at site +1 than at site +6.

To evaluate this, Z-scores were calculated using the mean ASC frequencies and standard deviations, as calculated from the aforementioned simulations, for each genome and plotted against GC3 content with the slope determined by linear regression of Z-score predicted by GC3 (**S1 Fig**). While for reasons unknown, significant negative relationships were observed between Z-score and genomic GC3 content at each position (Spearman’s rank: p < 2.2 x 10^-16^ for all positions; ρ = -0.61 at position +1, ρ = -0.65 at position +2, ρ = -0.51 at position +3, ρ = -0.45 at position +4, ρ = -0.41 at position +5, ρ = -0.46 at position +6), unexpectedly the negative slope is steeper at position +1 than position +6 (p = 5.2 x 10^-14^) contra to fail-safe hypothesis expectations. Indeed, there is a significant negative correlation between absolute gradient and distance from the primary stop (Spearman’s rank: p = 2.7 x 10^-3^, ρ = -1; **S2 Fig**), confirming that slope becomes flatter with 3’ distance. Using absolute stop codon frequency, rather than Z-score, confirms the same result (**S3 Fig**). These results additionally indicate that in GC-rich genomes, fail-safe ASCs are if anything avoided (note the Z scores control for dinucleotide content) and provide no support for the hypothesis that ASCs in closest proximity to the primary stop are more strongly preserved. This test comes with the caveat that the costs of stops further downstream may be relatively weak and thus failure to meet expectations is not necessarily strong evidence against the hypothesis. This being said, it is notable the trend is against our expectations.
