# Supplementary material for: In eubacteria, unlike eukaryotes, there is no evidence for selection favouring fail-safe 3’ additional stop codons
Source: PLoS Genet. 2019 Sep 17;15(9):e1008386. doi: 10.1371/journal.pgen.1008386 (PMC6764699; doi:10.1371/journal.pgen.1008386)
Supplement: S2 Text — (DOCX) [file pgen.1008386.s019.docx]

**S2 Text. Supporting text for S1 Table and S2 Table.**

Do we find no evidence for ASC enrichment because genomes specifically remove ASCs at a higher rate than chance? Perhaps switches from non-stop to stop occur at a lower rate than chance, and hence ASCs are a difficult evolutionary solution to stop codon readthrough? Further to the whole-UTR analysis in the main manuscript, we investigate both of these possibilities through analysing codon switches from stop to non-stop, and vice versa, in all downstream codon positions separately (**S1 Table**). Additionally, we look at a triplet of mollicute genomes to analyse TAA, TGA and TAG separately (**S2 Table**).
