# Supplementary material for: In eubacteria, unlike eukaryotes, there is no evidence for selection favouring fail-safe 3’ additional stop codons
Source: PLoS Genet. 2019 Sep 17;15(9):e1008386. doi: 10.1371/journal.pgen.1008386 (PMC6764699; doi:10.1371/journal.pgen.1008386)
Supplement: S3 Text — (DOCX) [file pgen.1008386.s020.docx]

**S3 Text. Supporting text for S4 Fig.**

Selection for termination efficiency is thought to be highest in HEGs due to the notion that the net effect of readthrough is a function of the number of translation events any given transcript is subject to. If the fail-safe hypothesis of ASCs is true, we therefore expect ASC frequencies to be significantly higher in HEGs than LEGs. This, however, does not seem to be the case. Standardised differences in ASC frequency for each genome [(HEGs – LEGs)/mean(HEGs + LEGs)] were calculated and are presented in **S4 Fig**. There were no significant differences between the ASC frequencies of HEGs and LEGs at any position (Wilcoxon signed-rank tests, p = 0.40 for position +1, p = 0.68 for position +2, p = 0.62 for position +3, p = 0.57 for position +4, p = 0.83 for position +5, p = 0.77 for position +6), suggesting that either expression level has no influence over the negative effects of readthrough or ASCs do not significantly affect the ability of a transcript to avoid these consequences.
