# Supplementary material for: In eubacteria, unlike eukaryotes, there is no evidence for selection favouring fail-safe 3’ additional stop codons
Source: PLoS Genet. 2019 Sep 17;15(9):e1008386. doi: 10.1371/journal.pgen.1008386 (PMC6764699; doi:10.1371/journal.pgen.1008386)
Supplement: S4 Text — (DOCX) [file pgen.1008386.s021.docx]

**S4 Text. Supporting text for S5 Fig.**

Are stop codons enriched when we allow for +4T enrichment? To compensate for the detected +4T enrichment, we returned to our initial null simulation experiment. This time we considered only +4T containing genes and adjusted our Markov models such that only +4T-containing genes were produced via simulation. Sequences were generated such that thymine was the first base 100% of the time, with following nucleotides selected according to dinucleotide frequencies. Therefore, these +4T-containing simulated sequences produced a null model appropriate for comparison with the +4T-containing genes from real genomes. In acknowledgement of a possible weak signals identified at position +1 (see main paper) and position +2 in HEGs (see **Fig 3** of the main paper)**,** we consider only these two positions. The null neutral expectation was that there is no difference between the ASC frequencies of the real genomes and simulated sequences. To assess this, we calculated Z-scores and completed binomial tests. Given our simulated sequences are built upon dinucleotide content alone we expect a random distribution of ASCs, thus our null expectation is a 50:50 split of positive and negative Z-scores. We find there to be significant variation from this ratio at both positions in all genes (Binomial tests: 34/644 Z > 0, p < 2.2 x 10^-16^ for position +1; 67/644 Z > 0 at position +2). These results are repeated in HEGs (Binomial tests: 1/22 Z > 0, p = 1.1 x10^-05^ for position 1; 4/22 Z > 0, p = 4.3 x10^-03^ for position 2) and LEGs (Binomial tests: 5/21 Z > 0, p = 0.026 for position 1; 3/21 Z > 0, p = 1.4 x10^-03^ for position 2). The result at position +1 in LEGs however does not survive multi-test correction (p > 0.05/2). As with the original simulations, in contrast to the prediction of enrichment per the fail-safe hypothesis, we note that deviation is due to under usage of ASCs (note the rarity of instances of Z > 0).

As before, we next looked at the proportion of genomes showing significant deviation from null (|Z| > 1.96). In this instance, the null expectation of the binomial test is no longer 50:50, rather that 95% of genomes will not be significantly deviated and 5% will. In all genes, there no significant deviation from this ratio at position +1 (Binomial test: 36/644, p = 0.4693) but significant deviation at position +2 (Binomial test: 0/644, p = 7.0 x 10^-15^). Closer examination indicates that significant difference at position +2 is due to under enrichment (Binomial test, alternative = ‘lower’: 0/644 Z > 1.64, p = 4.5 x 10^-05^ at position +1 and position +2). In HEGs and LEGs, there are no significant deviations from null (|Z| > 1.96).

The direction and magnitude of deviation from null was once again considered using the calculation of Z-scores. As discussed in the analysis of our previous simulations, the fail-safe hypothesis predicts resistance to GC pressure at position +1 and thus a flat slope when Z-score is plotted against genomic GC3. We repeat this analysis with our +4T-controlled null model (**S5 Fig**). Consistent with our original simulation-based analysis, we find Z-scores to be negatively correlated with genomic GC3 when considering all genes at position +1 (Spearman’s rank: ρ = -0.31, p < 2.2 x 10^-16^) and position +2 (Spearman’s rank: ρ = -0.19, p = 1.6 x 10^-06^). It therefore appears that ASCs are in fact avoided rather than enriched, consistent with our initial simulation experiment. No such negative relationships are found in HEGs and LEGs at either position (Spearman’s rank: p > 0.05), however low Z-score magnitudes (|Z| < 1.96) indicate that ASCs frequencies defy the fail-safe prediction of enrichment at the very least.
