# Supplementary material for: In eubacteria, unlike eukaryotes, there is no evidence for selection favouring fail-safe 3’ additional stop codons
Source: PLoS Genet. 2019 Sep 17;15(9):e1008386. doi: 10.1371/journal.pgen.1008386 (PMC6764699; doi:10.1371/journal.pgen.1008386)
Supplement: S5 Text — (DOCX) [file pgen.1008386.s022.docx]

**S5 Text. Supporting text for S6 Fig.**

Building on the observation that there may be a preference for fifth site thymine or cytosine in +4T-containing genes, we look at fifth site nucleotide frequencies. +4T-containing genes were extracted and fifth site nucleotide frequencies were calculated and compared (**S6 Fig**). Consistent with the enrichment of TC and TT-starting codons, there is preference for either thymine or cytosine when considering all genes. Fifth site T and C are both found in significantly higher frequency than the next most common nucleotide in TAA-terminating genes (Wilcoxon signed-rank tests: T > A, p < 2.2 x 10^-16^; C > A, p < 2.2 x 10^-16^), TGA-terminating genes (Wilcoxon signed-rank tests: T > A, p < 2.2 x 10^-16^; C > A, p = 4.2 x 10^-08^), and TAG-terminating genes (Wilcoxon signed-rank tests: T > A, p < 2.2 x 10^-16^; C > A, p = 3.7 x 10^-15^). Interestingly, in HEGs a fifth site T is preferred over C in all three groups (Wilcoxon signed-rank test: p = 3.7 x 10^-3^ in TAA-terminating genes; p = 6.2 x 10^-3^ in TGA-terminating genes; p = 0.034 in TAG-terminating genes), suggesting fifth site T is most optimal. In LEGs, there is no significant difference between any of the nucleotides at the fifth site of +4T-containing TAA-terminating genes (Kruskal-Wallis: χ = 7.503, p = 0.057). Adjusting for Bonferroni correction (p > 0.05/3), thymine is not present in significantly higher frequency than the next highest base in TGA-terminating LEGs (T > G – W = 130.5, p = 0.026) or in TAG-terminating LEGs (T > A – W = 114, p = 0.11).

The above test doesn’t control for GC pressure and may thus reflect an excess of AT rich genomes in our sample. To address this, we compare frequencies of TC or TT-starting codon frequency at position +1 to the average frequency of the respective codons between positions +1 to +6. In agreement with our frequency plots, we find TC-starting codons to be significantly enriched at position +1 (Wilcoxon signed-rank test: p < 2.2 x 10^-16^), and thus fifth site cytosine to be enriched in +4T-containing genes. However, we unexpectedly find no enrichment of TT-starting codons compared to null (Wilcoxon signed-rank test: p = 0.26). As this result is not consistent with our expectation, we cannot rule out the possibility that enrichment arises merely due to GC nucleotide pressure. Consistent with this we find that TT-codon usage at position +1 decreases with genomic GC3, whereas TC-codon enrichment increases (**S6 Fig**). This proposes a hypothetical model where fifth site thymine is favoured at low GC, and cytosine at high GC in +4T-containing genes.
