# Supplementary material for: In eubacteria, unlike eukaryotes, there is no evidence for selection favouring fail-safe 3’ additional stop codons
Source: PLoS Genet. 2019 Sep 17;15(9):e1008386. doi: 10.1371/journal.pgen.1008386 (PMC6764699; doi:10.1371/journal.pgen.1008386)
Supplement: S6 Text — (DOCX) [file pgen.1008386.s023.docx]

**S6 Text. Supporting text for S7 Fig.**

We acknowledge the limitations of LOESS modelling, which include those relating to the arbitrary nature of kernel/span function, and therefore validate the LOESS result with a different test design. Mollicute ASC frequencies were compared to GC-matched TT11 genomes (**S7 Fig**). In TT4 genomes, only TAA and TAG are used for chain termination. Hence, as TGA functions as a stop codon in TT11 genomes, it is expected under the fail-safe hypothesis that TGA frequency 3’ of the primary stop in TT4 genomes should be consistently lower than that in TT11 genomes. For each mollicute genome analysed, TT11 genomes of GC3 content within 3.5% were selected. The ASC frequencies of these genomes were calculated, and averages were calculated for each position. We find TGA to be underrepresented at positions +3 and +5 only (Wilcoxon signed-rank tests: p = 0.11 for position +1; p = 0.15 for position +2; p = 1.5 x 10^-3^ for position +3; p = 0.70 for position +4; p = 6.8 x 10^-4^ for position +5; p = 0.11 for position +6). This result is consistent with our LOESS analysis.
