# Supplementary material for: In eubacteria, unlike eukaryotes, there is no evidence for selection favouring fail-safe 3’ additional stop codons
Source: PLoS Genet. 2019 Sep 17;15(9):e1008386. doi: 10.1371/journal.pgen.1008386 (PMC6764699; doi:10.1371/journal.pgen.1008386)
Supplement: S7 Text — (DOCX) [file pgen.1008386.s024.docx]

**S7 Text. Supporting text for S3 Table.**

Prior mollicutes analysis (see **main paper** and **S4 Fig**) agrees with the hypothesis that TGA is underused in 3’ domains when it isn’t employed as a stop codon, compared with its usage in genomes of similar GC content when it can function as a stop. Whilst it remains possible that other codons may also be under-used in these genomes, for other reasons, our hypothesis does predict TGA to be among the most strongly under-enriched. We thus investigated all 64 codons using the aforementioned LOESS methodology and ranked them by their one-tailed Wilcoxon signed-rank test p-value (**S3 Table**). We find TGA to be the 25^th^ most under-enriched codon at position +1, 20^th^ at position +2, 4^th^ at position +3, 49^th^ at position +4, 2^nd^ at position +5, and 16^th^ at position +6. Instead, we find codons CCG (1^st^ at positions +1, +4, +6), GTG (2^nd^ at position 1, 3^rd^ at positions +4 and +6), and TAT (1^st^ at position +2, 2^nd^ at position +3, 4^th^ at position +1) among the more commonly underrepresented codons at specific positions. It therefore appears premature to presume that there is something special about TGA selection in bacterial 3’ UTRs relating to translational termination.
