# Supplementary material for: In eubacteria, unlike eukaryotes, there is no evidence for selection favouring fail-safe 3’ additional stop codons
Source: PLoS Genet. 2019 Sep 17;15(9):e1008386. doi: 10.1371/journal.pgen.1008386 (PMC6764699; doi:10.1371/journal.pgen.1008386)
Supplement: S8 Text — (DOCX) [file pgen.1008386.s025.docx]

**S8 Text. Supporting text for S8 Fig, S4 Table, and S5 Table.**

Given that the vast bulk of our evidence argues against ASCs functioning as fail-safe stop codons, we predict that there is no reason to maintain relative codon usage downstream of the primary stop. In contradiction, we find striking similarities between position +0 (primary stop) and positions +1 to +6 (**S8 Fig**). Surprisingly, we find that trends in TGA and TAG usage remains clearly decoupled despite their equal GC content (**S4 Table)**.

This result implies that stop codon usage at the primary stop is the same as usage at 3’ positions. However, this would not necessarily be the case if we were to find that relative codon usage is also maintained across the two other reading frames. We find this to be true (**S5 Table**), and hence question the validity of current hypotheses that release factor abundance explains the decoupling of TGA and TAG usage.
