# Supplementary material for: In eubacteria, unlike eukaryotes, there is no evidence for selection favouring fail-safe 3’ additional stop codons
Source: PLoS Genet. 2019 Sep 17;15(9):e1008386. doi: 10.1371/journal.pgen.1008386 (PMC6764699; doi:10.1371/journal.pgen.1008386)
Supplement: S9 Text — (DOCX) [file pgen.1008386.s026.docx]

**S9 Text. Supporting text for S6 Table.**

Our proposed hypothesis of gene shortening via the conversion of candidate stop codons (those that are upstream of the primary stop and are one-point mutation away from being a stop codon) to stop codons predicts that HEGs are longer in nucleotide length than LEGs. We consider only preliminary tests to indicate whether this may be true at a genomic level. Specifically, we assess the correlation between nucleotide length and protein abundance (**S6 Table**). Indeed, 18/22 demonstrate a negative trend of which 14/22 are significant.
