# Supplementary material for: In eubacteria, unlike eukaryotes, there is no evidence for selection favouring fail-safe 3’ additional stop codons
Source: PLoS Genet. 2019 Sep 17;15(9):e1008386. doi: 10.1371/journal.pgen.1008386 (PMC6764699; doi:10.1371/journal.pgen.1008386)
Supplement: S10 Text — (DOCX) [file pgen.1008386.s027.docx]

**S10 Text. Supporting text for S7 Table, S8 Table, and S9 Table.**

For the identification of ASC enrichment in eukaryotic genomes we apply three methodologies – Z-score deviation from dinucleotide-controlled null (**S7 Table**), Chi^2^ with dinucleotide-controlled null (**S8 Table**), and Chi^2^ with the Adachi and Cavalcanti null (**S9 Table**). Please find the detailed results of these analyses for each genome over the next few pages.
