# Supplementary material for: In eubacteria, unlike eukaryotes, there is no evidence for selection favouring fail-safe 3’ additional stop codons
Source: PLoS Genet. 2019 Sep 17;15(9):e1008386. doi: 10.1371/journal.pgen.1008386 (PMC6764699; doi:10.1371/journal.pgen.1008386)
Supplement: Attachment — Submitted filename: Reviewer comments PLOS_v6.docx [file pgen.1008386.s028.docx]

**Reviewer #1:**

“This is an interesting study demonstrating that eubacteria do not seem to contain additional stop codons down-stream from the primary stop codon of coding sequences. This pattern is in contrast to patterns observed in certain eukaryotes, whose 3' sequences downstream of stop codons are enriched in further stop codons.

The manuscript is carefully done and comprehensive. I have no concerns about the data analysis. However, I do have one substantive comment. In yeast, I'm aware that stop codon read through can be regulated via the yeast prion [PSI+] (see e.g., your Ref. 6 (Drummond and Wilke 2019), Fig. 3B, and references therein). I'm not sure whether a similar mechanism exists in ciliates. In any case, the [PSI+] state leads to extensive read through, much more than is observed under normal physiological conditions, and I could imagine that it would require extensive additional stop codons to be managed. If no such mechanism exists in eubacteria, then the selection pressure for 3' downstream stop codons would be much reduced.

I don't expect the authors to resolve this issue in their manuscript, but I think it would be warranted to add a paragraph or two about [PSI+] to the discussion. Currently, this mechanism isn't mentioned at all, and that seems like a major omission to me.”

We agree that this was an important omission – thank you for bringing to our attention. The idea of prion-like activity influencing read-through rates is now mentioned in the Abstract, Author summary, and considered in more detail in the discussion.

“Minor comment:

- Throughout, please verify that all figure elements are sized appropriately so they are legible. Specifically, the legends in Fig. 7 are definitely too small.”

All figures have been updated and verified by PACE (per the request of the journal).

**Reviewer #2:**

“This paper is well-written, except that it lacks scientific rigor both in building up the case and in drawing conclusions. I will give an example in each.

In building up the case, the authors stated that “adenine enrichment at the fourth coding sequence residue in bacterial genes promotes translation termination following a frameshift event at the initiating ATG that allows an out-of-frame stop codon to be read (9,10)”. I expect to see experimental evidence, but there isn’t any. The two papers cited in support of the claim are quite speculative. Two groups of scientists saying that something is possible does not equal to make that something an actuality.”

We agree that the language used when describing bioinformatic observations of ‘error-proofing’ selection – specifically adenine enrichment at the fourth coding residue of ATG start codons – was lacking experimental verification. We have hence reworded this sentence and others to reflect better the solidity with which certain observations are supported. We hope the referee agrees that our new wording is more appropriate.

“In making conclusions, the author stated that “Contra to the predictions of the hypothesis we find: there is paucity, not enrichment, of ASCs downstream”. This is not correct. I retrieved some compilation I did a long time ago on Escherichia coli that recorded the additional stop codon (ASC) occurrence downstream of the annotated codon (24 bases immediately downstream of the annotated codon). A stop codon is much more likely to occur immediately following the annotated codon than several triplets away. There is a highly significant negative correlation between the presence of a stop codon and the distance downstream from the annotated codon. I have verified this with the most recent E. coli genome. This finding is indeed what one would expect from the fail-safe mechanism. If fail-safe mechanism is to work well, it is better to have it right after the annotated stop codon than many based away downstream from it.

I could offer more examples, but I believe that this is sufficient for the authors.”

We considered this test but, given prior evidence in yeast, don’t consider it robust enough. While we agree that the evidence that the reviewer describes could be consistent with the fail-safe hypothesis (if fail-safe mechanism is to work well, it might be better to have it closer to the annotated stop codon), it isn’t good enough to reject the hypothesis should it not be found. Importantly, in yeast the enrichment is seen at site three exclusively so a linear regression of stop density with distance could fail to capture an effect. Moreover, unless one removes the first codon the test could be prone to false positives given the +4U effect.

Nonetheless, to address the referees concerns we have attempted to repeat the analysis, first using E. coli and then all of our T11 genomes. We consider only genes with >30 nucleotide 3’ intergenic space selected. 3’ UTR sequences (30 nucleotides downstream) were extracted for each eligible gene and the frequency of in-frame stop codons at each codon position calculated. ASC frequencies were plotted against codon position with both Spearman’s rank / Pearson’s R calculated. We do not find a significant negative relationship between ASC frequency and 3’ distance from the annotated primary stop (left figure). The weak tendency is destroyed on removing the first codon, whose stop enrichment can be explained by +4T/U effects (as we detail): the right-hand figure is as flat as a pancake.

**E. coli (including position +1)	E. coli (excluding position +1)**

Spearman’s: p-value = 0.3363, rho = -0.3666667	Spearman’s: p-value = 0.8401, rho = -0.0952381

Pearson’s: p-value = 0.2654, cor = -0.4160104	Pearson’s: p-value = 0.9886, cor = -0.006073923

We have replicated this across all 644 TT11 genomes. If anything, the effect overall goes the other way – stops avoided close to the primary stop (note both rho and slopes are right shifted towards positive not the predicted negative values). We use sign tests to verify that the median in each case is greater than 0 (Rho incl. pos +1, p = 8.882 x 10^-16^; Rho excl. pos +1, p = 6.661 x 10^-16^; Slope incl. pos +1, p < 2.2 x 10^-16^; Slope excl. pos +1, p = 1.998 x 10^-14^).

We repeat this test for our set of 68 single-celled eukaryote genomes and find no evidence for a median significantly deviated from 0 in any case (Sign tests; Rho incl. pos +1, p = 0.9063; Rho excl. pos +1, p = 0.1753; Slope incl. pos +1, p = 0.9063; Slope excl. pos +1, p = 0.05681).

We confirm that significant ASC enrichment (against at least one of our nulls) appears at one or more codon positions in 36 of these eukaryotic genomes. Restricting our analysis to enriched genomes only, we do not find a significant skew towards negative rho (Sign tests; Rho incl. pos +1, p = 0.87; Rho excl. pos +1, p = 0.16), nor towards negative slope (Sign tests; Slope incl. pos +1, p = 1; Slope excl. pos +1, p = 0.13). Hence, we remain unconvinced that this test is necessarily a good prediction of the fail-safe hypothesis.

Instead, stimulated by the referee’s considerations, we consider a new test. Given prospect of positional enrichment at different sites in different genomes, we ask how commonly we find genomes possessing ASC enrichment at one or more positions (from +2 to +6). This we think is a safer test of the referee’s logic. We find that bacteria are significantly under-represented to what would be expected by null chance, consistent with our prior finding that they are avoided. Please refer to the section ‘Lack of ASC enrichment in bacterial genomes reveals a discrepancy with single-celled eukaryotes’.

**Reviewer #3:**

“The authors have addressed here a very important question i.e. whether there exists ‘additional stop codons (ASC)’ as a ‘fail-safe’ mechanism for ‘stop codon read-through’ in eubacteria. Contradictory to the common belief they find that there is no enrichment (but, rather lack) of ASCs downstream, the correlation of ASC frequency is negative in relation to GC content of the genome.

While the work is highly interesting, I have a major criticism i.e. the lack of proper control for the method they have used. I elaborate the concern here. The authors have investigated the occurrence of ASCs by comparing with a dinucleotide controlled null. The logic behind this is not very clear.”

We agree that there could be greater clarity in explaining our dinucleotide-controlled null model, and this has now been addressed in the methods section. Furthermore, we have considered two other modes of testing against a null and find both give the same result as before – avoidance of downstream stops in bacteria.

“Since the whole conclusion is dependent on this primary analysis, the authors must provide results with positive and negative controls. It is well established that the eukaryotic genomes contain ASCs downstream the genes. The authors must validate their method by estimating Z score using the same method with equivalent number of eukaryotic genomes. Then the additional results can be accepted.”

Thank you for this valuable suggestion. We have added a new section to the results where we compare bacteria and eukaryotes using three methods: the Z-score methodology, a new Chi^2^ methodology and by the method used previously in ciliates. We extend the number of eukaryotic genomes tested for ASC enrichment with our set of 68 species. We find that all three methods give the same result – many fewer species of bacteria with ASC enrichment than single celled eukaryotes. We hope that the referee agrees that this thoroughly addresses the need for a robust eukaryote-bacteria comparison. Please see the new section: ‘Lack of ASC enrichment in bacterial genomes reveals a discrepancy with single-celled eukaryotes’.

“I also wonder why the analysis is restricted to only 600 genomes, while more than 5000 eubacterial genome sequences are available.”

We filter the full EMBL bacterial genome set to include just one genome per genus over 500,000bp. This helps reduce biases attributable to phylogenetic non-independence: evidence from many hundred E. coli samples are not independent data points. We consider our sample to be broad but not oversampled. The size restriction is to both remove plasmids and incomplete genomes but also to ensure a decent sample size of 3’ UTRs in all genomes (after filter). An explicit phylogenetic correction is also done in the species triplet analyses. These considerations are more fully explained in the methods section.

“There are mistakes in formatting the in-text references (e.g. page 19 - (Major, et al. 2002; Korkmaz, et al. 2014; Wei and Xia 2017)), which should be corrected.”

Errors in the in-text references have been fixed.
